# Menstrual Hygiene Management—Knowledge, Attitudes, and Practices Among Female College Students in Bhutan

**DOI:** 10.3389/frph.2021.703978

**Published:** 2021-08-27

**Authors:** Tashi Tshomo, Mongal Singh Gurung, Safieh Shah, Julita Gil-Cuesta, Peter Maes, Rinchen Wangdi, Jamba Tobden

**Affiliations:** ^1^Department of Public Health, Ministry of Health, Thimphu, Bhutan; ^2^Policy and Planning Division, Ministry of Health, Thimphu, Bhutan; ^3^The Medical Department, Médecins Sans Frontiéres, Operational Center Brussels, Brussels, Belgium; ^4^The Institute for Gross National Happiness Studies, Royal University of Bhutan, Thimphu, Bhutan

**Keywords:** knowledge, attitudes, practice, menstruation, menstrual cycle, dysmenorrhea, taboo

## Abstract

**Background:** Girls and women face substantial menstrual hygiene management (MHM) challenges in low- and middle-income countries. These challenges are related to inadequate knowledge and insufficient water, sanitation, and hygiene (WASH) facilities. Currently, the literature on MHM among college-attending women in Bhutan is scarce. We aimed to explore the knowledge, attitudes, and practices (KAP) of female college students from all the 10 government colleges of Bhutan, documenting the conditions of available MHM facilities, from August to September 2018.

**Methods:** A cross-sectional KAP survey was conducted with a random sample of female students from all years and a random sample of MHM facilities at each college and hostel. A questionnaire was adapted from a similar study conducted with school students in Bhutan. Socio-demographics, overall KAP findings, and differences in KAP between first and final year students were analyzed; college and hostel toilets were self-reported and directly observed.

**Results:** In the survey, 1,010 participants completed the self-administered questionnaire. The comprehensive knowledge of menstruation was found to be low (35.5%) among participants. Half of the participants (50.3%) reported their mother as the source of information, and 35.1% of the participants agreed that women should not enter a shrine during menstruation. It was also reported that approximately 4% of median monthly pocket money was spent on the absorbents, and 96.9% of absorbents were wrapped before disposal. Half of the participants (55.1%) reported that their daily activities were affected due to menstruation, and 24.2% of the female students missed college due to dysmenorrhea. One-fifth of the participants (21.3%) reported unavailability of water in college, 80.1% of the participants reported absence of soap for hand washing, and 24.1% described no bins for disposal. The participants also reported that in 33.7% of hostel toilets, the door locks were missing. The direct observations also had similar findings.

**Conclusions:** Female students living in hostels during college years lose considerable resources during their formative years of learning, such as time, energy, and money, due to issues of menstruation management. Although the overall understanding of menstruation was low, the MHM practices of our participants scored highly, and the vast majority of them asked for a platform to discuss menstruation. Despite some agreement with menstrual taboos (e.g., visiting shrine), only 5.1% of the participants were uncomfortable conversing about MHM. Improved public health knowledge, psychosocial/medical support, and WASH infrastructure with freely available menstrual products could lead to more effective MHM practices among female college students.

## Introduction

Menstrual hygiene management (MHM) refers to the specific hygiene and health requirements of girls and women during menstruation, such as the knowledge, information, materials, and facilities needed to manage menstruation effectively and privately (1). Inadequate water, sanitation, and hygiene (WASH) facilities, particularly in public places such as college campuses and hostels, can pose a major challenge to women and girls regarding the safe disposal of the used menstrual materials and the ability to wash their hands (2, 3).

Multiple systemic reviews and meta-analyses of studies of MHM behavior and practices in low and middle-income countries show that women and school girls report substantial health, as well as social challenges, when it comes to managing their menstruation (4–7).

Menstruation has been surrounded by misperceptions and taboos in society causing reluctance to talk about it (5, 8, 9). Studies show that beliefs regarding menstruation are deep-rooted, and girls describe the onset of menarche as a shocking experience, a curse from God, or even as punishment for the sins of their ancestors (1, 10–12). One such study in Bhutan demonstrated this even in schools (13).

Studies conducted in low- and middle-income countries such as Bhutan, India, Saudi Arabia, and Iran found that girls received information on menstruation mainly from their mothers, (11, 12, 14–16) who tended to focus on activities to be avoided due to traditional taboos (17, 18). Taboos lead to socially imposed restrictions, such as exclusion from daily prayers, avoiding certain foods, performing fasting ceremonies, avoiding touching holy books or flowers, and even preventing them from entering a kitchen or a temple (8, 18–21) as the blood of menstruation is considered “dirty” (14). The failure to fully acknowledge the physical reality of women has a range of serious impacts alongside with experiences of shame (22).

Studies have found a lack of safe and clean hygiene facilities, which leads to unsatisfactory opportunities to clean external genitalia and to change stained absorbents (8, 23). The existing evidence highlights either a lack of disposal facilities for absorbents or inadequate and poorly maintained means of disposal (24, 25). This affects the education of girls: They miss their classes during menstruation due to fear of staining, shame, ridicule by their peers, menstrual cramps, or the lack of facilities to manage their menstrual hygiene privately (10–12, 26). This has led many girls and women to dispose of absorbents and pads with routine waste in toilets or in open spaces (5, 8, 19).

In countries near Bhutan, China, and Bangladesh, tailored education sessions have been found to improve knowledge on menstruation and practices among adolescent girls (27, 28).

In Bhutan, research studies with school-going adolescent girls and rural women (9, 11, 17) have identified gaps in knowledge, inadequate facilities, and socio-cultural barriers for practicing hygienic MHM. However, little is known about this among women who attend college. Although college-going girls or women have not been studied, there is a societal assumption that they must be educated enough to have a good understanding of MHM. This may not be true. As the main source of menstrual information seems to be mothers in Bhutan, not checking the true knowledge, attitudes, and practices (KAP) of college women could have far-reaching implications if they continue to transmit incorrect information and stigma to their daughters (10, 19).

This study offered a unique opportunity to identify existing gaps in MHM among government colleges that need to be addressed throughout Bhutan. The findings should guide the Ministry of Health (MoH) in Bhutan to make recommendations for improving MHM in these colleges.

Thus, our objective was to describe the KAP of female college students on MHM and evaluate the physical conditions of college WASH facilities for basic MHM.

## Materials and Methods

### Design

A cross-sectional KAP survey was conducted, and physical observation studies of MHM facilities were carried out in all 10 government colleges of Bhutan from August 8 to September 13, 2018.

### Setting

There are two universities that provide modern education in Bhutan: the Royal University of Bhutan (RUB) and the Khesar Gyalpo University of Medical Sciences of Bhutan (KGUMSB). As of 2018, there were 11,259 students pursuing various courses in these tertiary institutions and women made up 46% of the total enrollment (29). The students at these colleges (both male and female) are admitted based on their merit, independent of where they live. The majority of students do not live at home during their college years and are provided with a full government scholarship and accommodation in college hostels, independent of their socioeconomic status. Less than 20% stay outside the college campus either in rented private rooms or with their relatives.

### Population

The target population for this study was female students pursuing undergraduate courses in 2018 at eight colleges of RUB and two colleges of KGUMSB. The colleges and institutes that do not offer undergraduate courses were excluded. All female students who provided informed consent and were above the age of 18 years were included in the study.

### Sample Size

The sample size was estimated with an assumed percentage of absenteeism during menstruation to be 48% (11). At a 95% confidence level with a 5% acceptable margin of error in estimating the percentage of absenteeism during menstruation and a 90% response rate, estimates were calculated for three subgroups (first year, second year, and final year students). The final sample size was 1,280 students.

### Data Collection

The data collection team was composed of the principal investigator (PI) and trained female research assistants from the MoH, Bhutan, and each college, respectively. Data were collected during working hours at the colleges for the period of 1 month, from August 8 to September 13, 2018.

The participants were randomly selected from the total list of eligible students enrolled in the 10 colleges (sampling frame = 4,194). The sampling was proportional to the size of the college. The data collection team visited the 10 colleges with the list of selected students based on this sampling. A common venue was arranged, and selected participants were requested to gather and learn about the study. Informed consent was sought from each individual before they completed the self-administered questionnaire. Enough distance was maintained at the venue between each of the participants to ensure the privacy of their responses.

A self-reported questionnaire with multiple-choice responses was used to assess the KAP of the participants. This questionnaire was adapted from the KAP survey on MHM conducted among school-going girls by the Ministry of Education in Bhutan. Socio-demographic variables, such as age, religion, year of study, current place of residence, and the educational level of the mother, were included. The questions related to physiology, female anatomy, and menstrual hygiene were asked under the knowledge domain. The participants were categorized as having “comprehensive knowledge” if they knew correct answers to all the five questions under the knowledge domain. “Attitudes” were assessed using a rating scale from strongly agree, agree, disagree, and strongly disagree for questions on social and cultural beliefs. To ascertain “practices,” the participants were asked regarding the type of absorbents used, their cost, absenteeism, hygiene practices disposal, and whether there was a need for a platform to discuss MHM.

The study assessed MHM facilities through (i) responses to the KAP questionnaire by the participants about the toilet facilities in their hostels and at colleges and (ii) direct observation by our team that visited a cross-section of toilets at each of the 10 colleges. An observation checklist was formulated for the assessment. The observation was undertaken to corroborate the condition of the MHM facilities so that it was not based solely on the perception of the participants. For this, a checklist was used to assess MHM facilities in at least one toilet per college or hostel looking for soap, a dustbin, water, and a door with locks. The toilets nearest to the venue of data collection were chosen for observation.

A pilot study was conducted among the female postgraduate students at the Royal Institute of Management (RIM), an autonomous institute based in Thimphu, Bhutan, to trial the recruitment strategy and to evaluate the clarity of the questionnaire and informed consent prior to the study.

### Data Analysis

Frequencies and percentages of the socio-demographic characteristics of the study population and their KAP were reported. Costs of absorbents in USD (US dollars), means, SD, median, and interquartile range were reported for MHM practices according to variable type such as avoiding some foods, taking a bath, and cleaning genitals. The subgroup analysis by year in college (first, second, and final year) was carried out for KAP. The differences in knowledge and practices between first and final year students were assessed using the chi-square test. A comparison was made between first and final year students to establish an association between MHM and educational level. MHM facilities, such as hand washing facilities, soap, pad disposal bins, and lockable doors reported by students through KAP and observed by data collectors, were analyzed and compared.

The data were entered into EpiData Entry software version 3.1 and analyzed using Stata 15 (StataCorp. 2017. *Stata Statistical Software: Release 15*. College Station, TX: StataCorp LLC). Data were double-entered and validated by two data entry staff recruited and trained for this purpose.

### Ethics Approval and Consent to Participate

Approval to conduct the study was obtained from the Research Ethics Board of Health (REBH) (Approval no: Ref. No. REBH/Approval/2017/077) under the MoH in Bhutan and Médecins Sans Frontières (MSF) Ethics Review Board (MSF approval no ID 1776). Further, administrative clearances were obtained from the RUB and KGUMSB college administrations. Individual written informed consent was obtained from each participant.

## Results

### Socio-Demographic Characteristics

Out of 1,280 female students approached, 1,021 completed the questionnaires (response rate of 79.8%) and 1,010 were included (11 were minors, and informed consent from their parents or legal guardians could not be obtained). Their mean age calculated was 20.7 years (SD 1.71). The proportions by college year were roughly divided into three (Table 1). More than 90.7% of the participants were Buddhist. Other socio-demographic characteristics are reported in Table 1.

**Table 1:** Socio-demographic characteristics of female college students in the study (*n* = 1,010).

**Socio-demographic characteristics**	**Participants**	
	**Number**	**Percentage**
Mean age of participants (Mean ± SD)	20.68 (±1.71)	
**Years in College**		
First	349	34.6
Second	340	33.7
Final (Third and Fourth)	321	31.8
**College**		
College of Language and Cultural Studies	166	16.4
College of Natural Resources	108	10.7
College of Science and Technology	61	6.0
Faculty of Nursing and Public Health	55	5.5
Faculty of Traditional Medicine	9	0.9
Gaeddu College of Business Studies	148	14.7
Jigme Namgyel Engineering College	67	6.6
Paro College of Education	89	8.8
Samtse College of Education	92	9.1
Sherubtse College	215	21.3
**Accommodation** ^**a**^		
Hostel	905	89.8
Rented Rooms	75	7.4
Parents	22	2.18
Others (other relatives)	6	0.6
**Religion** ^**b**^		
Buddhist	913	90.7
Hindu	74	7.4
Christian	15	1.5
Others (Kirat, Manav, etc)	5	0.5
**Hometown by Region** ^**c, e**^		
Western	268	27.5
Central	292	30.0
Eastern	413	42.5
**Mother's Educational Level** ^**d**^		
No education	537	53.4
Non-formal education (for adults)	196	19.5
Primary (PP to 6)	118	11.8
Secondary (7 to 12)	109	10.8
Degree and above	34	3.4
Monastic	7	0.7
Others (Secondary+Certificate/Diploma)	5	0.5

*MHM, Menstrual Hygiene Management; SD, Standard Deviation ^a^Missing, 2; ^b^Missing, 3; ^c^Missing, 37; ^d^Missing, 4; ^e^Bhutan has three regions. The eastern region has the highest number of registered voters*.

### Knowledge of Menstruation and Source of Information

Table 2 shows that 35.4% of the students knew the correct answer to all five questions and that 11 (1.1%) students did not know the correct answer to a single question. The proportion of students having comprehensive knowledge was higher among final year students compared to the first year (38.6% vs. 31.0%, *p*-value 0.037). The main source of information on menstruation was mothers for half (50.3%) of the students, with the rest reporting teachers (19.5%), friends (14.4%), sisters (14.3%), and others (1.9%).

**Table 2:** Number and percentage of female college students with appropriate knowledge about MHM (*n* = 1,010).

**Knowledge**	**Participants with correct knowledge** ***n*** **(%)**				***P*-value^***l***^**
	**Overall**	**1st Year**	**2nd Year**	**3rd Year**	
What is menstruation^*a, g*^	967 (96.4)	326 (94.8)	330 (97.4)	311 (97.2)	0.115
Cause of menstruation^b, h^	863 (87.4)	292 (86.4)	292 (88.0)	279 (87.7)	0.608
Organ from where menstrual blood come^c, i^	743 (76.2)	237 (72.3)	256 (76.9)	250 (79.6)	0.029*
Normal menstruation duration for normal person^*d, j*^	834 (82.6)	278 (79.7)	278 (81.8)	278 (86.6)	0.017*
Interval between two menstrual cycle in days^*e, k*^	535 (53.0)	165 (47.3)	188 (55.3)	182 (56.7)	0.015*
**Combined**					
Comprehensive knowledge^*f*^	357 (35.4)	108 (31.0)	125 (36.8)	124 (38.6)	0.037*
Having correct knowledge on 1–4 questions	642 (63.5)	233 (66.8)	213 (62.6)	196 (61.1)	0.124
Incorrect answer to all five questions	11 (1.1)	8 (2.3)	2 (0.6)	1 (0.3)	0.028*^∧^

*MHM, Menstrual Hygiene Management. ^a^It's “Natural shedding of blood on monthly basis” and it's not (“a disease on monthly basis,” “Type of curse received by women,” “All of them,” “others,” and “Don't know”).*

*^b^It's Hormones and it's not (“Curse of God,” “Caused by diseases,” “Others,” and “Don't know”).*

*^c^It's Uterus and it's not (bladder, abdomen, others, and “don't know”).*

*^d^It's 3–7 days.*

*^e^It's 28–42 days.*

*^f^Individual having the correct knowledge on all fives questions.*

*^g^Missing, 7; ^h^Missing, 22; ^i^Missing, 35; ^j^Missing, 16; ^k^Missing, 43.*

*^l^Chi-square test was calculated for the difference between first and final years.*

*There is some evidence against the null hypothesis of no difference.

*^∧^Mid-P exact test*.

### Perceptions and Attitudes Toward Menstruation

**Figure 1:**
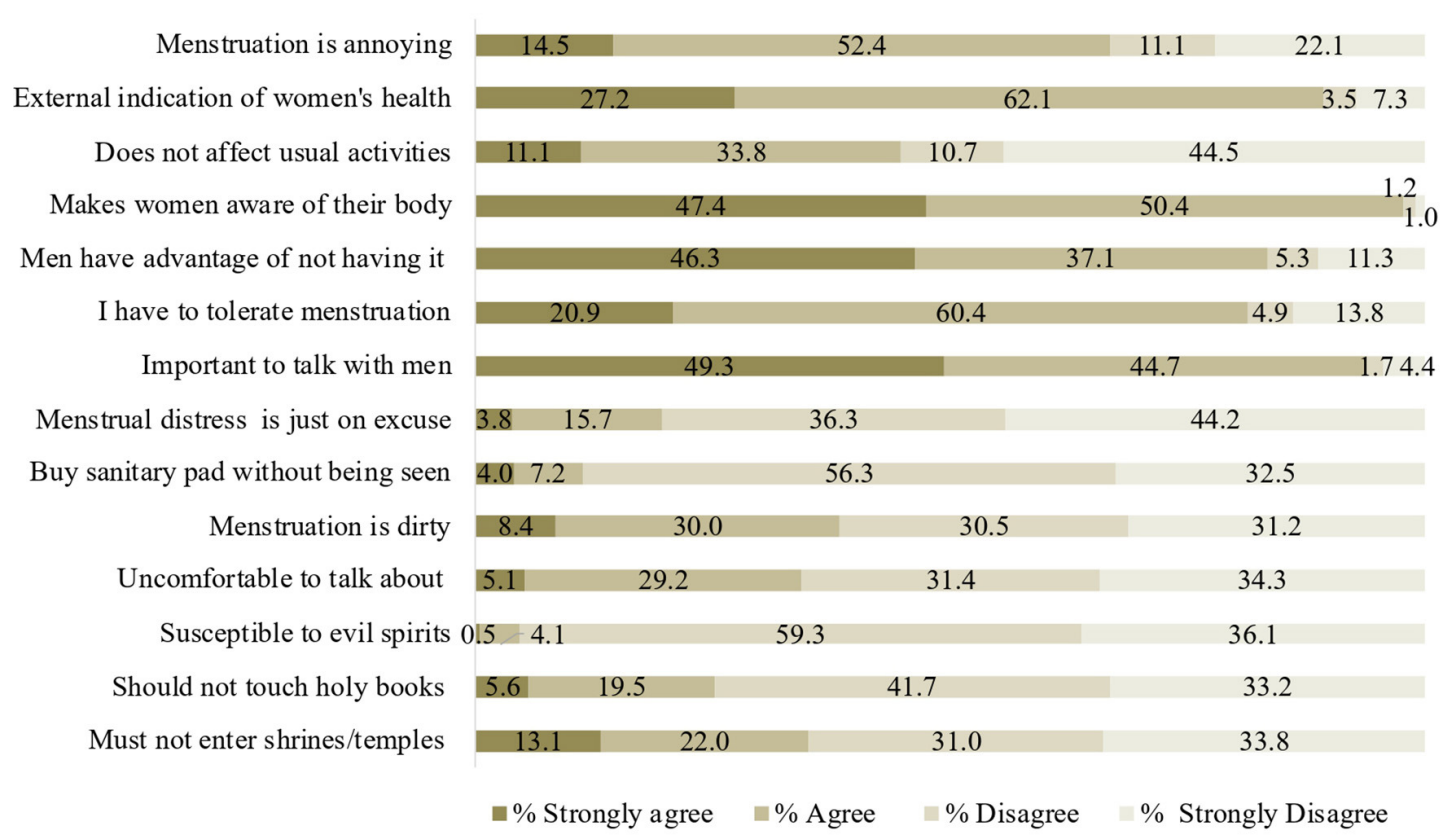
Attitudes toward menstruation among female college students in Bhutan (*n* = 1,010).

Nearly half (44.9%) of the participants agreed that menstruation affects their daily activities, and 94.0% of the participants strongly agreed on the importance of talking about menstruation (Figure 1). More than 45% of the participants strongly agreed that men have the advantage of not having menstruation. Only 5.1% of the participants strongly agreed with the uncomfortable feeling to address MHM in a conversation. About one-third (35.1%) of the participants agreed that women should not enter a shrine when menstruating.

### Practices During Menstruation

The median amount of money spent on absorbents by college students was Ngultrum (Nu) 80 (1.14USD) per month (IQR Nu. 60), and the median amount of pocket money students received was Nu 2000 (28.6 USD) per month (IQR Nu. 2000). Among the participants, 98.6% took a bath during menstruation and 96.9% wrapped used absorbents before disposing them. Among the participants, 24.2% reported missing classes during menstruation (Table 3).

**Table 3:** Practices related to MHM among female college students in all the 10 government colleges of Bhutan, 2018 (*n* = 1,010).

**MHM related practices**	**Yes** ***n*** **(%)**				***P*-value^***f***^**
	**Overall**	**1st Year**	**2nd Year**	**3rd Year**	
Miss college during menstruation^*a*^	241 (24.2)	61 (17.9)	83 (24.6)	97 (30.7)	<0.001*
Avoid some food during menstruation^*b*^	312 (31.1)	110 (31.6)	91 (26.8)	111 (35.1)	0.337
Take bath during menstruation^*c*^	955 (95.0)	331 (95.1)	324 (95.3)	300 (94.3)	0.654
Clean genitals during menstruation^*d*^	997 (99.2)	344 (98.9)	339 (99.7)	314 (99.1)	0.817^∧^
Wrap pad before disposing^*e*^	971 (96.8)	338 (97.7)	325 (95.9)	308 (96.9)	0.509

*MHM, Menstrual Hygiene Management.*

*^a^Missing, 14; ^b^Missing, 7; ^c^Missing, 4; ^d^Missing, 5; ^e^Missing, 7.*

*^f^Chi-square test was calculated for the difference between first and third years.*

*There is a strong evidence against the null hypothesis of no difference.

*^∧^Mid-P exact test*.

The mean number of days missed during menstruation was 1.4 days per month (SD 0.8). Reasons for absenteeism were pain (86.1%), afraid of staining (5.6%), and feeling uncomfortable (4.4%).

### Menstrual Hygiene Management Facilities in Colleges and Hostels

Tap water in college toilets was reported to be missing by 21.3% of the participants. Indeed, the data collection team found that over one-third (38.9%) of college toilets did not have water when they visited (Table 4).

**Table 4:** Status of menstrual hygiene management facilities in all the 10 government colleges of Bhutan, 2018.

**Characteristics of MHM facilities**	**Self-reported (*****n*** **=** **1,010)**		**Observed (*****n*** **=** **18)**	
	**Yes** ***n* (%)**	**No** ***n* (%)**	**Yes** ***n* (%)**	**No** ***n* (%)**
**College toilets**				
Lockable doors for MHM^*a*^	597(61.0)	382 (39.0)	14 (77.8)	1 (5.6)
Water for MHM^*b*^	775 (78.7)	210 (21.3)	9 (50.0)	7 (38.9)
Soap for hand washing^*c*^	199 (19.9)	803 (80.1)	2 (11.1)	14 (77.8)
Bin for pad disposal^*d*^	759 (75.9)	241 (24.1)	9 (50.0)	6 (33.3)
**Hostel toilets**				
Lockable doors for MHM^*e*^	652 (66.3)	332 (33.7)	13(72.2)	1 (5.6)
Water for MHM^*f*^	784 (81.0)	184 (19.0)	12 (66.7)	6 (33.3)
Soap for hand washing	Not asked	Not asked	6 (33.3)	12 (66.7)
Bin for pad disposal^*g*^	805 (81.2)	187 (18.85)	10 (55.6)	8 (44.4)

*^a^Missing, 31; ^b^Missing, 25; ^c^Missing, 8; ^d^Missing, 10; ^e^Missing, 26; ^f^Missing, 42; ^g^Missing, 18*.

Soap for hand washing was missing in 77.8% of the college facilities during observation, and 80.1% of the participants also reported the same. The conditions were similar in their hostels.

### Platform for Discussion and Education on MHM

Among the participants, 916 (91.6%) said that there was a need for platforms to talk about MHM. The preferred platforms were sessions on MHM within the colleges (95.7%) followed by social media groups (26.3%).

## Discussion

Our cross-sectional study found inadequate comprehensive knowledge of MHM among female college students in Bhutan. A majority of women (>50%) appeared to be quite knowledgeable although a few students did not know the answer to any of the “knowledge” questions about menstruation. The scores for “practices” were found to be better than for “knowledge,” almost all students reported that they bathed during menstruation and disposed products properly. Only one-quarter still agreed with beliefs such as not entering shrines or not touching holy books during menstruation and menstruation being dirty. In spite of this, almost all students expressed their interest to talk further on MHM. There was a notable lack of MHM facilities observed at the colleges that correlated with reports from the students.

We expected a higher level of education of the participants to correspond with a higher knowledge of menstruation. This was true, despite an overall low score on comprehensive knowledge. This finding was further confirmed by comparing sub-groups. Final year students had higher comprehensive knowledge compared to first year students, possibly due to the influence of peers. Similar evidence from a study of Saudi nursing students linked an increase in education level to increased knowledge of menstruation, although no reason was given for the association (14). The effect of the practices getting better with years spent in college could be attributed to peer support. Studies in China and Bangladesh found adequate and accurate information on menstruation, which is important to improve practice on MHM (27, 28, 30).

The proportion of the female college students agreeing with socio-cultural beliefs, such as not entering a shrine or menstruation being dirty, is small but similar to a study among school girls in Bhutan (11). This may indicate that beliefs do not change with an increase in educational level, although our study did not find the association between the two. A similar study in Nepal found that cultural beliefs lead girls to practice self-imposed restrictions like not entering temples or joining prayer ceremonies (18). These beliefs and taboos remained as menstruation was not discussed and these perceptions are passed through generations (20, 24). However, our findings show how women that are having at least a little knowledge about menstruation may not endorse such taboos. Gender issues seem to be an important result of this study due to the sense of injustice felt as most participant say that men have an advantage over women of not having menstruation. While menstruation is a healthy and integral part of female identity, the cultural message of menstruation to be gross, troubling, or shameful has created a dominant narrative of menstruation as a negative, troubling, and problematic experience for those who menstruate (22, 31). It indicates that there is a need to provide an adequate information package that will normalize menstruation, change attitudes, and end negative social norms (24, 31).

Approximately half of our participants claimed that menstruation affected their usual activities. The majority rated pain as the main reason for absenteeism from college. This is comparable to the assessment conducted among school-going girls in Bhutan (11). A similar study in Mumbai found that the most common problems faced during menstruation were menstrual cramps. In the survey, 44.6% of school girls said that menstrual cramps affected their usual activities and 53.6% of them agreed that women feel more tired than usual during menstruation (11). Absenteeism was noted with an increase in education in our study. However, further analyses would be needed to explore the correlation between absenteeism and educational level along with other variables that were not included in our data collection. Absenteeism due to menstrual cramps may affect the academic performance of a student. Studying a correlation between academic performance and menstrual cramps was beyond the scope of our study, and future research studies in this area would be interesting.

Another key finding of this study was inadequate MHM facilities like water, soap, and bins for disposal of absorbents in both hostels and college toilets, compromising the ability of the students to practice proper hygiene. These findings have been corroborated by a systematic review carried out in low- and middle-income countries where women and girls were unable to undertake their preferred menstrual practices due to inadequate MHM infrastructure (6, 32, 33). Lack of safe spaces for MHM may affect the health and dignity of women and girls (32). Issues of access to facilities and attitudes go hand in hand in causing exclusion, stigma, and disadvantage (22). The participants expressed a strong wish for platforms to talk about menstruation in their college, and only a small proportion said they were uncomfortable in discussing it. Studies in China, Bangladesh, and elsewhere have shown that educational sessions have enhanced knowledge, promoted a more positive attitude, and improved practices such as managing menstrual cramps (6, 15, 28). Significant increases in menstrual knowledge and confidence among women were observed following a more open discourse (15).

### Strengths and Limitations

This study had some strengths and limitations. First, college women in Bhutan are chosen based on merit and come from different socioeconomic classes. This improves its generalizability. Second, the study sample was taken from the female college population in all Bhutanese government colleges; therefore, it is generalizable at a national level. Third, we used direct observation of WASH facilities to triangulate with the reports from the students.

The main limitation of our study is that it was a self-administered survey, possibly subject to social desirability bias, with no indication of how participants interpreted the questions. We mitigated this limitation considerably by conducting a pre-test to adjust the questions and also by having the research team stay at the site during data collection to clarify any questions for the participants. The questions on “know about menstrual hygiene” and “infection due to poor MHM” might have overestimated the actual knowledge of the participants on menstrual hygiene, since the participants may have avoided replying to questions, which they did not know. Finally, although we observed WASH facilities in each of the colleges, we could not include the associations between the MHM facilities and the practices of the participants in our analyses, as the observations of MHM facilities were not sufficiently representative.

The study has the following implications: First, increased educational sessions in schools and colleges could improve MHM practices. The focus should be on evidence-based hygiene practices and demystifying false beliefs that limit the participation of women and girls in education and other socio-cultural activities, such as eating certain foods (1, 18, 20). Second, adequate physical facilities to practice MHM are crucial in improving hygiene practices. This should be followed by timely monitoring of these facilities (34). Sensitization of men may be a logical outcome. Men on campus and in the community could ensure adequate menstrual supplies are available for female students (35). Also, colleges could ensure that a healthcare provider is available who can help women when they feel unwell, treat the side effects of menstruation, and assess their urogenital health in case of infections. The college and management should take immediate action to ensure the availability of clean running water and soaps, bins with lids for disposal of sanitary bins, and secure, lockable doors in the toilet facilities.

### Conclusions

This study of KAP related to MHM found significant knowledge and belief gaps but some encouraging practices among female students in government colleges of Bhutan. It also revealed important inadequate physical and psychosocial facilities to support the practices of these students, leading to absenteeism. There are clear ways forward to tackle these problems, and we encourage college administrations to address them.

## Data Availability Statement

The datasets generated and/or analyzed during the current study are not publicly available due to sensitivity of disaggregated data for each colleges. However, anonymized datasets are available from corresponding author on reasonable request.

## Ethics Statement

The study was reviewed and approved by Research Ethics Board of Health (REBH) (Approval no: Ref. No. REBH/Approval/2017/077) under the Ministry of Health in Bhutan and Médecins Sans Frontières (MSF) Ethics Review Board (MSF approval no ID 1776). The participants provided their written informed consent to participate in this study.

## Author Contributions

TT, SS, PM, MG, JT, and RW conceptualized the study. TT collected and cleaned the primary data. TT and JG-C conducted the analysis and interpretation of the data. TT, JG-C, and SS drafted the manuscript. TT, MG, JG-C, PM, and SS revised the manuscript. All authors approved the final manuscript.

## Conflict of Interest

The authors declare that the research was conducted in the absence of any commercial or financial relationships that could be construed as a potential conflict of interest.

## Publisher's Note

All claims expressed in this article are solely those of the authors and do not necessarily represent those of their affiliated organizations, or those of the publisher, the editors and the reviewers. Any product that may be evaluated in this article, or claim that may be made by its manufacturer, is not guaranteed or endorsed by the publisher.

## Abbreviations

KAP: Knowledge Attitude and Practice
KGUMSB: Khesar Gyalpo University of Medical Sciences of Bhutan
MHM: Menstrual Hygiene Management
MSF: Medécins Sans Frontières
Nu: Ngultrum (Bhutanese Currency)
REBH: Research Ethics Board of Health
RUB: Royal University of Bhutan
SD: Standard Deviation
SORT IT: Structured Operational Research and Training Initiative
STROBE: Strengthening the Reporting of Observational Studies in Epidemiology
WASH: Water and Sanitation
WHO: World Health Organization.
