# Supplementary material for: Menstrual Hygiene Management—Knowledge, Attitudes, and Practices Among Female College Students in Bhutan
Source: Front Reprod Health. 2021 Aug 27;3:703978. doi: 10.3389/frph.2021.703978 (PMC9580629; doi:10.3389/frph.2021.703978)
Supplement: Supplementary file 1 [file Table_1.DOCX]

**Supplementary material 1: Questionnaire**

Menstrual Hygiene Management (MHM) in the government colleges of Bhutan KAP Survey 2018

Q1.How old are you (**AGE** in completed year): ……………

Q2. Your permanent address (**DISTRICT**): ……………………

Q3. Which **YEAR** student are you? (Tick one):

🞎 First

🞎 Second

🞎 Third

🞎 Others (specify): ………………………………………..

Q4.What is your **RELIGION**:

🞎 Buddhist

🞎 Hindu

🞎 Christian

🞎 Others (specify): ...........................................

Q5. Where do you **STAY** in college?

🞎 College hostels (including self-catering)

🞎 Rented private rooms

🞎 With parents or relatives

🞎 Others (specify): ......................................................

Q6. With whom do you usually **STAY** **during** **winter or summer vacation**? (Tick one)

🞎 Both parents (Mother and father)

🞎 Mother only

🞎 Father only

🞎 Households without any elder female member(s)

🞎 Households with elder female member(s) other than mother

🞎 Alone

🞎 Others (specify): ............................

Q7. What is your **MOTHER’S EDUCATION LEVEL**? (**Optional**)

🞎 No Education

🞎 NFE

🞎 Primary (PP to Class VI)

🞎 Secondary (Class VII to Class XII)

🞎 Certificate or Diploma

🞎 Degree+

🞎 Monastic

🞎 Others (Specify): ..............................

**A. SOCIO-DEMOGRAPHIC CHARACTERISTICS**

Q8. What is menstruation?

🞎 A disease on monthly basis

🞎 Natural shedding of blood on monthly basis

🞎 Type of curse received by women

🞎 All of them

🞎 Others

🞎 Don’t know

Q9. What is the cause of menstruation?

🞎 Curse of God

🞎 Caused by diseases

🞎 Hormones

🞎 Others

🞎 Don’t know

Q10. From which organ does the menstrual blood come from?

🞎 Uterus

🞎 Bladder

🞎 Abdomen

🞎 Others

🞎 Don’t know

Q11a. Do you know the duration of normal menstruation for a normal person?

🞎 Yes

🞎 Don’t know

Q11b. What is duration of normal menstruation for a normal person?

🞎 ............................. (in days)

🞎 Don’t know

Q.12. What is interval between two menstrual cycles? (in days)

1. .............days

2.dont know

Q.13. Did anyone tell you about menstruation before your onset of menstruation?

1. Yes

2. No

3. I don't remember

Q.14. From whom did you get information regarding menstruation (circle several responses if that was the case)

1. Mother

2. Teacher

3. Friends

4. Sisters

5. television

6. Internet

7. Others

Q.15. Do you know what menstrual hygiene is?

1. Yes

2. No

Q.16. Do you know about infections due to poor menstrual hygiene?

1. Yes

2. No

**B. KNOWLEDGE**

| **C. ATTITUDES (TICK YOUR RESPONSE** in one box against each question**)** | | | | | | | |
| --- | --- | --- | --- | --- | --- | --- | --- |
|  | **Attitudes** | **Strongly agree** | **Agree** | **Strongly Disagree** | **Disagree** | **Don’t know** | **Choose not to answer** |
| 17 | Women must not enter shrines/temples while having menstruation |  |  |  |  |  |  |
| 18 | Women in menstruation are susceptible to get possessed by evil spirits |  |  |  |  |  |  |
| 19 | Menstruation does not affect usual activities |  |  |  |  |  |  |
| 20 | I have to tolerate menstruation |  |  |  |  |  |  |
| 21 | Men have real advantage of not having monthly period |  |  |  |  |  |  |
| 22 | Menstruation makes women more aware of their body |  |  |  |  |  |  |
| 23 | The recurrent monthly flow is external indication of women's health |  |  |  |  |  |  |
| 24 | Women complaining on menstrual distress is just an excuse |  |  |  |  |  |  |
| 25 | It is important to talk about menstrual period with men |  |  |  |  |  |  |
| 26 | Menstruation is dirty |  |  |  |  |  |  |
| 27 | Menstruation is annoying |  |  |  |  |  |  |
| 28 | It is important to buy sanitary pad without being seen |  |  |  |  |  |  |
| 28 | It is uncomfortable for women to talk about menstruation |  |  |  |  |  |  |
| 29 | Women should not touch holy books during menstruation |  |  |  |  |  |  |

Q.30 What is the main absorbent material do you use during period/menstruation? (Circle **only one** response)

1. sanitary pad

2. napkin/cloth or towel

3. reusable sanitary pad

4. tampon

5. menstrual cup

6. cotton

7. tissue paper

8. Others

9. none of the above

Q.31 Why do you prefer?(circle **only one** response

1. Easily available

2.affordable

3.can be used longer

4.others

**D. PRACTICES**

Q.32 Which one do you prefer the most if given choice?(circle **only one** choice)

1. sanitary pad

2.napkin/cloth or towel

3. reusable sanitary pad

4.tampoon

5.menstrual cup

6.cotton

7.tissue paper

8.Others....................................

9.none of the above

Q.33 Do you receive pocket money?

1.Yes

2.No

Q.34 What amount of monthly pocket money do you receive?

Number( Nu) ......................

Q.35 What is the cost of absorbent you use per month/

Number(Nu) ..............

Q.36 If you are using reusable absorbent, how do you wash it? (circle **only one** response)

1. Only with water

2. with water and soap

3. others

Q.37. If you are using reusable absorbent, how do you dry it?

1. sunlight

2. inside the house

3. others

Q.38. how many times do you change cloth/pad in a day 1. once

2.two or more

Q.39 where do you dispose your pads?

1.pad disposal bin

2. drain

3.toilet

4.open field

5. routine waste

6.others(specify)....................................

Q.40 Do you wrap the pad before disposing? 1.Yes

2.No

Q.41Type of pad wraps used for disposing it

1.Papers

2.plastic wrap

3.no wraps

4.Others(specify)...........................

Q.42 Do you usually miss college during menstruation? If No skip to Q.45

1.Yes

2.No

Q.43. If Yes, how many days in a month do you miss college?

1. one day

2.two days

3. three days

4. Others (specify)……………..

Q.44 Reasons for missing college during menstruation?(May chose more than one response ) 1. afraid of staining

2. it cause pain

3. it makes me feel uncomfortable

4.no adequate toilets

5. no washing facilities

6.Others(specify).................................

Q.45. What remedies do you take during menstruation? 1. medication

2.Yoga/meditation

3.Prayers

4.thoroughly involve in activities

5.take rest

6.others (specify)..............................

Q.46 Do you avoid some food during menstruation? 1,Yes

2.No

Q.47 If yes, What food do you avoid during menstruation?

.............................

Q.48 Do you take bath during menstruation?

1.Yes

2.No

Q.49 If yes, how often?

1. Daily

2. only on first day

3. only on second day

4. only on last day

5. Others(others)……………………..

Q.50 Do you clean genital area during menstruation?

1.Yes

2. No

Q.51. If Yes, by what?

1. water and soap

2. only with water

3. towels

4. others (specify).............................

Q.52. Which of the following sanitary disposal facilities does your college have? (May choose more than one) 1. Burning places

2. Pit

3. Pad disposal bins

4. none

5. others (specify)................................

Q.53. Does your college have place for drying clothes?

1.Yes

2.No

.54 Does your college have soap for washing hands in the college toilets?

1.Yes

2.No

Q.55 Does your college toilets have bins identified for disposal of pads?

1. Yes

2. No

Q.56 If Yes, where is it located?

1. individual toilets

2. one in the common toilets

3. 3. Others(specify)

Q.57 . Does your hostels have bins identified for disposal of pads?

1. Yes

2. No

Q.58 If Yes, where is it located?

1. individual toilets

2. one in the common toilets

3.Others (specify)

Q59. Do college toilets have lockable doors for MHM?

1. Yes

2.No

Q.60 Does hostels toilets have lockable doors for MHM

1.Yes

2.No

Q.61 Does college toilets have water for MHM?

1.Yes

2.No

Q.62 Does hostels toilets have water for MHM?

1.Yes

2. No

Q. 63.Do you feel there is need for a platform to talk about MHM?

1.Yes

2.No

Q.64 what is the most preferred platform for you?

1. Social media group

2. IEC materials

3. Sessions on MHM in college

4. Others (specify)

***Thank you for participation!***
